# Case report: Local anesthesia with lidocaine infiltration for extended-release buprenorphine therapy

**DOI:** 10.3389/fpsyt.2025.1500799

**Published:** 2025-01-23

**Authors:** Pouya Azar, Jane J. Kim, Ella Rohani, Dayyon Newman-Azar, Matin Narimani, Jessica Machado, Victor W. Li

**Affiliations:** ^1^ Integrated Psychiatry, Pain, and Addiction Service, Vancouver General Hospital, Vancouver, BC, Canada; ^2^ Department of Psychiatry, University of British Columbia, Vancouver, BC, Canada

**Keywords:** opioid use disorder, lidocaine, extended-release buprenorphine, injection site pain, case series

## Abstract

**Background:**

Extended-release buprenorphine (BUP-XR) is a once-monthly subcutaneous injection for the treatment of opioid use disorder. Injection-site pain is a common adverse event reported with BUP-XR administration. Notwithstanding the advantages of BUP-XR, subjective pain and anxiety associated with injections can compromise patients’ willingness to receive treatment. Lidocaine is an amide-type agent and sodium channel blocker commonly used for local and regional anesthesia in various fields of medicine.

**Case presentation:**

We present two cases involving lidocaine infiltration to the induction phase of BUP-XR therapy in an outpatient setting. Prior to the intervention, 2 mL of 1% lidocaine was infiltrated subcutaneously at the sites of the planned needle insertion for a numbing effect. The following BUP-XR therapy was well tolerated by both participants and reported as a painless procedure.

**Conclusions:**

Lidocaine infiltration may be a feasible way to successfully initiate and provide BUP-XR therapy to those who may be deterred by injection-related risks. Our cases describe how lidocaine can be useful in mitigating injection-site pain and encouraging greater uptake, and in turn, greater retention in opioid agonist therapy.

## Introduction

Mortality rates due to opioid overdose remain alarmingly high in Canada. Since 2016, the country has seen over 44,000 opioid-related deaths and almost the same number of opioid-related hospitalizations (1). In 2023, a total of 8,049 apparent opioid toxicity deaths were reported, a number 7% higher than the same period in 2022 (1). Individuals with opioid use disorder (OUD) face an annual risk of death nearly 15 times higher than that of the general population, with overdose being their most common cause of death (2). Opioid receptor agonists, including methadone and buprenorphine, are the mainstays of pharmacological treatment for OUD (3, 4). However, despite their demonstrated efficacy, the need for daily oral dosing has been shown to have negative impacts on patient compliance, limiting the overall duration and success of therapy (5).

Extended-release buprenorphine (BUP-XR or Sublocade^®^) is administered monthly rather than daily through a subcutaneous injection in the abdomen (6, 7). Throughout the month, it provides a sustained release of buprenorphine from the injected depot, offering palpable benefits in terms of longer and more flexible dosing windows and reduced risk of diversion (8, 9). BUP-XR is indicated only for OUD patients who are clinically stabilized for at least 7 days on 8-24 mg of transmucosal buprenorphine to suppress opioid withdrawal symptoms. Due to its propensity to form a solid mass following subcutaneous administration, it further carries the risk of serious adverse reactions with inadvertent intradermal, intramuscular, or intravenous administration (10). Dosages recommended for the treatment of OUD include two initial 300 mg injections, followed by 100 mg injections for maintenance (11). However, patients established on long-term treatment with transmucosal buprenorphine may be directly transitioned to BUP-XR with only one or none of the higher loading doses for the first two months of treatment. In turn, maintenance doses may also be increased to 300 mg monthly for patients who do not demonstrate a satisfactory clinical response with the 100 mg dose (12). Several clinical trials have demonstrated the efficacy and safety of BUP-XR. In one randomized, double-blind, phase 3 clinical trial of adults with moderate to severe OUD, BUP-XR significantly increased abstinence from opioids and had higher medication satisfaction compared to placebo (13). The safety profile of BUP-XR was also found to be consistent with other buprenorphine formulations, all with the exception of injection-site reactions.

Injection-site pain (ISP) is a common treatment-emergent adverse event (TEAE) with BUP-XR administration. In a 12-month multicenter phase 3 study of 527 adults with moderate to severe OUD, ISP was reported by 13.2% of participants, with mean pain scores of 44.0 out of 100 within the first hour of injection (14). Another study reported ISP in 18.3% of its depot buprenorphine recipients following subcutaneous administration, most of which were graded as mildly intense (15). Pain, both experienced and anticipated, can reduce the acceptability of treatment in patients. In one focus group, cases of at least one patient declining future injections in light of ISP were noted by several providers of an outpatient addiction clinic (16). Patient endorsement of ISP was further described in qualitative studies to consist of soreness, bruising, and general unpleasantness adjusting to the lump in their abdominal tissue post-injection (17–19). Several factors related to the injection technique and composition of the solution can affect the sensation of pain in subcutaneous injections (20). Specific to BUP-XR, it is likely that the 19-gauge 5/8-inch needles used for BUP-XR administration may provoke more painful injections (12).

Local anaesthetic infiltration is frequently used to prevent pain prior to a surgery or procedure. Lidocaine, also known as lignocaine, is an amide-type agent and sodium channel blocker commonly used for local and regional anesthesia (21). By interacting with voltage-gated potassium and sodium channels, it is believed that lidocaine reduces the peak currents and suppresses the activation of neurons responsible for receiving sensory information about pain (22, 23). It has a superior safety profile compared to opioids and other analgesics and is utilised across a wide range of procedures in the fields of dermatology, dentistry, and otolaryngology (24, 25). Due to its short action onset of 2-5 minutes and duration of 1-2 hours, it is particularly suitable for use in outpatient and emergency department settings (24). Infiltration with lidocaine prior to BUP-XR administration may be a feasible option to minimize the pain experienced by patients. Though published evidence is scant, the use of either injectable or topical lidocaine as a method of pain relief has been described in several protocols for BUP-XR injections (26–29). However, the scope of the practice has largely been limited to clinics within the United States and exempt from dissemination in other countries like Canada, where it could benefit a much larger pool of patients.

To the best of our knowledge, there is yet to be a descriptive report on the effect of instilling lidocaine on the pain associated with BUP-XR injections. Notwithstanding the advantages of BUP-XR, subjective pain at the injection site can compromise patients’ willingness to receive injections and their overall compliance to treatment. Given the critical role of pain in the acceptability of treatment by patients, we hereby describe two successful cases of pain relief with lidocaine-infiltrated BUP-XR administration in patients with OUD. Written and verbal informed consent were obtained from both patients.

## Case description

### Case 1

A 61-year-old female presented to the outpatient Transitional Pain Clinic with chief complaints of chronic foot pain. She had a past medical history of mild OUD in sustained remission, nicotine use disorder, anxiety, and depression. She was treated with 8 mg of daily buprenorphine-naloxone for her OUD in the two months prior but had found this dose ineffective in reducing her risk of relapse. She expressed interest in starting BUP-XR therapy due to its ease of use and was assessed to be an eligible candidate. BUP-XR was believed to overall offer her better protection against both fatal and non-fatal overdose and serve as a viable option to address her chronic pain. Previous to intervention, her abdominal injection site was cleaned with ethanol swabs and injected with 2 mL of 1% lidocaine in saline. Around 1 mL was put into the subcutaneous space and the rest was used to form a small skin bleb. Minimal bleeding was observed, and injection pain was rated about 1 out of 10 in severity, with 0 being no pain. Preparedness for the following BUP-XR injection was tested by touching the bleb with the lidocaine needle for a sharp sensation, which the patient described as “not sharp at all”. This was followed by 300 mg of BUP-XR injected through the bleb into the subcutaneous space. She verbally reported a score of 0 out of 10 for the injection and that she did not even feel the needle go in. The patient left satisfied and wanting to return for a second injection for her OUD and chronic foot pain. In a follow-up phone consultation post one-month of treatment, she further described that the injection had caused her no issues with no visible signs of redness or infection. She also did not endorse using any extra opiates or having cravings.

### Case 2

A 45-year-old female diagnosed with opioid and methamphetamine use disorders presented to the emergency department at a tertiary care hospital in Vancouver, British Columbia with pneumonia. Her past medical history included anemia, brain aneurysms, depression, psoriasis, and pain in her back, bilateral hip, and throughout her body. She was using opiates for the past two years and reported smoking approximately 1 gram of fentanyl per day. Prior to admission, she had been treated with methadone and buprenorphine-naloxone but reported both as unsuccessful due to her missing doses and having to restart. She expressed readiness to quit opioid use and was assessed to be an appropriate candidate for BUP-XR due to the ease of monthly administration. Her abdominal injection site was cleaned with ethanol swabs and pre-administered with 2 mL of 1% lidocaine injection. Once local anesthesia was achieved and confirmed by touch, the skin was tented up and 300 mg of BUP-XR was injected into the subcutaneous space. No other adjuvant therapy was asked for or provided to the patient. The patient reported a pain score of 0 for both injections. No signs of redness or necrosis were observed on or around the wound. In a follow-up consultation after three weeks, she reported that BUP-XR therapy had helped her greatly and that she had managed to cut down on her fentanyl use to approximately half a gram per day.

## Discussion

Extended-release buprenorphine therapy by means of subcutaneous injections is used routinely for the treatment of OUD. Subcutaneous injections can cause pain upon skin puncture, which can add to patient anxiety and reluctance to engage in further treatment. This case series demonstrates success among two OUD patients pre-administered with 2 mL of 1% lidocaine to mitigate the pain associated with BUP-XR therapy. Both patients successfully completed a painless induction process, and although long-term outcomes on retention were not reported, showed willingness to continue their treatment.

ISP upon subcutaneous injections are common occurrences in BUP-XR therapy. They are often described to be transient with severity levels ranging from mild to moderate (30). However, regardless of their rated intensity, subjective pain can be a determinant for adherence in many of those with needle anxiety or low tolerances for pain. One qualitative study documented how patients were apprehensive of BUP-XR therapy due to worries that it might be painful or their general dislike for needles. Others endorsed more specific fears that the injection might create a hematoma or act as a negative reminder of their previous injecting history (31). This suggests that, for select patients, the injection route of administration and associated fear can result in the avoidance of an efficacious therapy. There is an ongoing need to remain alert to how extended-release and other novel formulations accord with patient preferences and how treatment-associated anxiety may implicate their care. Further, given the significance of pain in affecting patient perceptions of treatment acceptability and their anticipated adherence, more research attention should be directed toward strategies to reduce treatment-emergent pain. Documented strategies thus far have ranged from providing ibuprofen, ice packs, and even lollipops as a method of distraction from the injection (32, 33). The main advantages of infiltrating lidocaine relative to other non-invasive approaches include its fast onset, precise delivery on the injection site, and reliable anesthesia (24). Alternative approaches such as topical anesthetics, ice packs, and vibration anesthesia devices may carry a slower and duller effect but still hold benefit for those who are averse to all forms of injection. Establishing a standardized protocol for lidocaine infiltration would assist practitioners’ clinical decision making among the various pain management approaches, allow for consistency in administration, and permit comparisons between different clinical settings.

Our case series has shown that infiltration with lidocaine can help mitigate ISP in an acute care outpatient setting. Feedback from patients indicated a painless procedure that easily facilitated the initiation of BUP-XR therapy. Moreover, the addition of a lidocaine injection did not significantly extend the duration of the patients’ stay nor added to the clinical workload. Ensuring that BUP-XR injections are well tolerated on site can limit the chances of its recipients being lost to follow-up or abandoning future treatment. Further investigation of the appropriateness of the lidocaine infiltration in other settings, such as inpatient or community-based, is warranted.

Other clinicians have reported that the addition of epinephrine to lidocaine can prolong the duration of anesthetic effect and reduce toxicity (34). We chose to administer lidocaine without the addition of epinephrine to avoid increasing any pain on the injection site as well as possible blanching effect on the skin. We further ensured that the first injection with lidocaine was painless with a small, 30-gauge needle (24). No tissue site swelling or necrosis were observed in either of our cases. In the future, it would be interesting to compare how solutions of lidocaine versus lidocaine with epinephrine affect pain upon injections.

Induction and long-term retention in opioid agonist therapy (OAT) remain critical challenges for public health systems. The rate of discontinuation is high among all forms of therapy; not limited to BUP-XR. In a retrospective data linkage study of 220,474 OAT dispensations in British Columbia, Canada, it was found that more than 40% of episodes initiated with methadone, buprenorphine, and slow-release oral morphine did not complete induction (35). In another study of individuals who transitioned from sublingual to extended-release buprenorphine, 48% of patients discontinued treatment after three months (36). The factors that impede the access and retention to OAT go beyond ISP and span across health care providers, patients, and organizational systems (37). Greater recognition of these barriers is necessary to promote client entry and receipt of OUD treatment, especially in times when rates of opioid-related deaths are higher than ever.

## Conclusion

This case series demonstrates that local infiltration with lidocaine can remove the pain of subcutaneous BUP-XR injections. Such an approach may be a feasible way to successfully initiate and retain those who may have been deterred from BUP-XR by the pain of injections and associated needle anxiety. Optimizing OAT satisfaction has become particularly important in recent years, with growing rates of attrition among its patients (Tahsin et al., 2022). Improving patients’ overall experience with subcutaneous injections may encourage greater uptake of BUP-XR, and in turn, result in greater treatment retention. As with all case series, we are limited by its small sample size of two, which limits its generalizability. More research, including larger randomized controlled trials to compare lidocaine and other local anaesthetic agents in injectable OAT, is needed to improve patient care.

## Data Availability

The original contributions presented in the study are included in the article/**Supplementary Material**. Further inquiries can be directed to the corresponding author/s.

## References

[1] Government of Canada. Opioid- and Stimulant-related Harms in Canada. Ottawa: Public Health Agency of Canada (2024). Available at: https://health-infobase.Canada.ca/substance-related-harms/opioids-stimulants/ (Accessed September 20, 2024).

[2] Degenhardt L Bucello C Mathers B Briegleb C Ali H Hickman M. Mortality among regular or dependent users of heroin and other opioids: a systematic review and meta-analysis of cohort studies. Addict Abingdon Engl. (2011) 106:32–51. doi: 10.1111/j.1360-0443.2010.03140.x 21054613

[3] Mattick RP Breen C Kimber J Davoli M. Methadone maintenance therapy versus no opioid replacement therapy for opioid dependence. Cochrane Database Syst Rev. (2009) 2009:CD002209. doi: 10.1002/14651858.CD002209.pub2 12519570

[4] Mattick RP Breen C Kimber J Davoli M. Buprenorphine maintenance versus placebo or methadone maintenance for opioid dependence. Cochrane Database Syst Rev. (2014) 2. doi: 10.1002/14651858.CD002207.pub4/full PMC10617756 24500948

[5] Wood P Opie C Tucci J Franklin R Anderson K. A lot of people call it liquid handcuffs” – barriers and enablers to opioid replacement therapy in a rural area. J Subst Use. (2019) 24:150–5. doi: 10.1080/14659891.2018.1523968

[6] British Columbia Centre on Substance Use (BCCSU). A guideline for the clinical management of opioid use disorder (2023). Available online at: https://www.bccsu.ca/wp-content/uploads/2023/12/BC-OUD-Treatment-Guideline_2023-Update.pdf (Accessed September 20, 2024).

[7] Lofwall MR Walsh SL Nunes EV Bailey GL Sigmon SC Kampman KM. Weekly and monthly subcutaneous buprenorphine depot formulations vs daily sublingual buprenorphine with naloxone for treatment of opioid use disorder: A randomized clinical trial. JAMA Intern Med. (2018) 178:764–73. doi: 10.1001/jamainternmed.2018.1052 PMC6145749 29799968

[8] Chappuy M Trojak B Nubukpo P Bachellier J Bendimerad P Brousse G. Prolonged-release buprenorphine formulations: Perspectives for clinical practice. Therapies. (2020) 75:397–406. doi: 10.1016/j.therap.2020.05.007 32499082

[9] Scott R Aboud A O’Gorman T. Long-acting injectable buprenorphine - “best practice” opioid agonist therapy for Australian prisoners. Australas Psychiatry Bull R Aust N Z Coll Psychiatr. (2022) 30:498–502. doi: 10.1177/10398562211059086 34852654

[10] Rosenthal RN. Novel formulations of buprenorphine for treatment of opioid use disorder. Focus. (2019) 17:104–9. doi: 10.1176/appi.focus.20180043 PMC6527006 31975965

[11] Soyka M Franke AG. Recent advances in the treatment of opioid use disorders–focus on long-acting buprenorphine formulations. World J Psychiatry. (2021) 11:543–52. doi: 10.5498/wjp.v11.i9.543 PMC8474991 34631459

[12] Sublocade (buprenorphine extended-release) injection, for subcutaneous use, CIII. Food and Drug Administration (FDA) (2022). Available at: https://www.sublocade.com/Content/pdf/prescribing-information.pdf.

[13] Haight BR Learned SM Laffont CM Fudala PJ Zhao Y Garofalo AS. Efficacy and safety of a monthly buprenorphine depot injection for opioid use disorder: a multicentre, randomised, double-blind, placebo-controlled, phase 3 trial. Lancet. (2019) 393:778–90. doi: 10.1016/S0140-6736(18)32259-1 30792007

[14] Andorn AC Haight BR Shinde S Fudala PJ Zhao Y Heidbreder C. Treating opioid use disorder with a monthly subcutaneous buprenorphine depot injection: 12-month safety, tolerability, and efficacy analysis. J Clin Psychopharmacol. (2020) 40:231. doi: 10.1097/JCP.0000000000001195 32282418 PMC7188268

[15] Lintzeris N Dunlop AJ Haber PS Lubman DI Graham R Hutchinson S. Patient-reported outcomes of treatment of opioid dependence with weekly and monthly subcutaneous depot vs daily sublingual buprenorphine: A randomized clinical trial. JAMA Netw Open. (2021) 4:e219041. doi: 10.1001/jamanetworkopen.2021.9041 33970256 PMC8111483

[16] Reddy IA Audet CM Reese TJ Peek G Marcovitz D. Provider perceptions toward extended-release buprenorphine for treatment of opioid use disorder. J Addict Med. (2024) 18(5):540–5. doi: 10.1097/ADM.0000000000001320 PMC11446660 38829032

[17] Cheng A Badolato R Segoshi A McDonald R Malone M Vasudevan K. Perceptions and experiences toward extended-release buprenorphine among persons leaving jail with opioid use disorders before and during COVID-19: an in-depth qualitative study. Addict Sci Clin Pract. (2022) 17:4. doi: 10.1186/s13722-022-00288-4 35093164 PMC8800291

[18] Johnson B Flensburg OL Capusan AJ. Patient perspectives on depot buprenorphine treatment for opioid addiction – a qualitative interview study. Subst Abuse Treat Prev Policy. (2022) 17:40. doi: 10.1186/s13011-022-00474-2 35614466 PMC9131643

[19] Neale J Parkin S Strang J. How do patients feel during the first 72 h after initiating long-acting injectable buprenorphine? An embodied qualitative analysis. Addiction. (2023) 118:1329–39. doi: 10.1111/add.v118.7 36808168

[20] Usach I Martinez R Festini T Peris JE. Subcutaneous injection of drugs: literature review of factors influencing pain sensation at the injection site. Adv Ther. (2019) 36:2986–96. doi: 10.1007/s12325-019-01101-6 PMC6822791 31587143

[21] Bahar E Yoon H. Lidocaine: A local anesthetic, its adverse effects and management. Medicina (Mex). (2021) 57:782. doi: 10.3390/medicina57080782 PMC8399637 34440986

[22] Yang X Wei X Mu Y Li Q Liu J. A review of the mechanism of the central analgesic effect of lidocaine. Med (Baltimore). (2020) 99:e19898. doi: 10.1097/MD.0000000000019898 PMC7440315 32332666

[23] Wolff M Schnöbel-Ehehalt R Mühling J Weigand MA Olschewski A. Mechanisms of Lidocaine’s action on subtypes of spinal dorsal horn neurons subject to the diverse roles of Na+ and K+ Channels in action potential generation. Anesth Analg. (2014) 119:463. doi: 10.1213/ANE.0000000000000280 24892804

[24] Golzari SE Soleimanpour H Mahmoodpoor A Safari S Ala A. Lidocaine and pain management in the emergency department: A review article. Anesthesiol Pain Med. (2014) 4:e15444. doi: 10.5812/aapm.15444 PMC3961016 24660158

[25] Mao P Zhang Y Liu B Li Y Chang Y Zhu M. Effect and safety profile of topical lidocaine on post-surgical neuropathic pain and quality of life: A systematic review and meta-analysis. J Clin Anesth. (2024) 92:111219. doi: 10.1016/j.jclinane.2023.111219 37827033

[26] Boston Medical Center. Standing order for administration of injectable lidocaine prior to Injection of Long Acting Injectable Buprenorphine (LAIB) (2022). Available online at: https://www.addictiontraining.org/documents/resources/352_Boston_Medical_Center_Injectable_Lidocaine_LAIB_Protocol.pdf (Accessed September 10, 2024).

[27] Ganetsky VS. Clinical Updates on the Use of Injectable Medications for Opioid Use Disorder (MOUD). Cooper University Health Care. Available at: https://sites.rutgers.edu/mat-coe/wp-content/uploads/sites/473/2021/05/Clinical-Updates-on-the-Use-of-Injectable-Medications-for-Opioid-Use-Disorders-MOUD-05072021.pdf (Accessed September 10, 2024).

[28] Kan D. Long Acting Injectible Buprenorphine (LAI-BUP): An Introduction. Center for Care Innovcations. Available at: https://www.careinnovations.org/wp-content/uploads/Sublocade-Presentation_060820.pdf (Accessed September 10, 2024).

[29] San Mateo County Health. Comparison of Buprenorphine (BUP) long-acting subcutaneous injections . Available online at: https://www.addictiontraining.org/documents/resources/352_Boston_Medical_Center_Injectable_Lidocaine_LAIB_Protocol.pdf (Accessed September 10, 2024).

[30] Marsden J Kelleher M Gilvarry E Mitcheson L Bisla J Cape A. Superiority and cost-effectiveness of monthly extended-release buprenorphine versus daily standard of care medication: a pragmatic, parallel-group, open-label, multicentre, randomised, controlled, phase 3 trial. eClinicalMedicine. (2023) 66. https://www.thelancet.com/journals/eclinm/article/PIIS2589-5370(23)00488-1/fulltext (Accessed September 20, 2024). 10.1016/j.eclinm.2023.102311 PMC10692661 38045803

[31] Neale J Tompkins CNE McDonald R Strang J. Implants and depot injections for treating opioid dependence: Qualitative study of people who use or have used heroin. Drug Alcohol Depend. (2018) 189:1–7. doi: 10.1016/j.drugalcdep.2018.03.057 29857327

[32] Hassman H Strafford S Shinde SN Heath A Boyett B Dobbins RL. Open-label, rapid initiation pilot study for extended-release buprenorphine subcutaneous injection. Am J Drug Alcohol Abuse. (2023) 49:43–52. doi: 10.1080/00952990.2022.2106574 36001871

[33] Lancaster K Gendera S Treloar C Rhodes T Shahbazi J Byrne M. Tinkering with care: Implementing extended-release buprenorphine depot treatment for opioid dependence. Int J Drug Policy. (2024) 126:104359. doi: 10.1016/j.drugpo.2024.104359 38382354

[34] Strazar AR Leynes PG Lalonde DH. Minimizing the pain of local anesthesia injection. Plast Reconstr Surg. (2013) 132:675–84. doi: 10.1097/PRS.0b013e31829ad1e2 23985640

[35] Kurz M Min JE Dale LM Nosyk B. Assessing the determinants of completing OAT induction and long-term retention: A population-based study in British Columbia, Canada. J Subst Abuse Treat. (2022) 133:108647. doi: 10.1016/j.jsat.2021.108647 34740484 PMC9833672

[36] Stein MD VanNoppen D Herman DS Anderson BJ Conti M Bailey GL. Retention in care for persons with opioid use disorder transitioning from sublingual to injectable buprenorphine. J Subst Abuse Treat. (2022) 136:108661. doi: 10.1016/j.jsat.2021.108661 34801283

[37] Socías ME Dong H Wood E Brar R Richardson L Hayashi K. Trajectories of retention in opioid agonist therapy in a Canadian setting. Int J Drug Policy. (2020) 77:102696. doi: 10.1016/j.drugpo.2020.102696 32050143 PMC7577708

